# Role of Bone Morphogenetic Proteins-7 (BMP-7) in the Renal Improvement Effect of DangGui (*Angelica sinensis*) in Type-1 Diabetic Rats

**DOI:** 10.1093/ecam/nep167

**Published:** 2011-08-22

**Authors:** Ching-Hua Yeh, Chen-Kuei Chang, Kai-Chun Cheng, Ying-Xiao Li, Ying Wen Zhang, Juei-Tang Cheng

**Affiliations:** ^1^Institute of Medical Science, College of Health Science, Chang Jung Christian University, Kway Jen, Tainan 71101, Taiwan; ^2^Institute of Basic Medical Sciences and Department of Pharmacology, College of Medicine, National Cheng Kung University, Tainan City 70101, Taiwan; ^3^Department of Medical Research, Chi Mei Medical Center, Yung Kang City, Tainan Shan 71004, Taiwan; ^4^Department of Surgery, Mackay Memorial Hospital, Graduate Institute of Injury Prevention and Control, Taipei Medical University, Taipei City 10107, Taiwan; ^5^Department of Psychosomatic Internal Medicine, Kagoshima University Graduate School of Medical and Dental Sciences, Sakuragaoka, Kagoshima City, Japan; ^6^Department of Chinese with Western Medicine, Zhongnan Hospitial, Wuhan University, Wuhan 430071, China

## Abstract

Hyperglycemia induced reactive oxygen species (ROS) generation is believed as major factors leading to diabetic nephropathy (DN). DangGui (*Angelica sinensis*) is mentioned to show renal protective effect in combination with other herbs. Bone morphogenetic proteins-7 (BMP-7) is produced merit in protection of DN. The role of BMP-7 in DangGui-induced renal improvement is not clear. The present study investigated the effects of DangGui on renal functions, BMP-7 expression and the levels of ROS in streptozotocin (STZ)-induced diabetic rats and high glucose-exposed rat mesangial cells (RMCs). After 1- or 4-week treatment, DangGui improved renal functions and increased renal BMP-7 expression in diabetic rats. The BMP-7 expression in RMCs was reduced by high glucose treatment and this could be reversed by DangGui. Moreover, RMCs exposed to high glucose were expired by BMP-7 RNAi transfection but those cells remained alive by scramble transfection. Thus, we employed regular RMCs to knock down BMP-7 with RNAi and we found that DangGui increased BMP-7 expression in these RMCs. Direct activation of BMP-7 expression by DangGui could be considered. The results of DPPH assay, DHE stain and lucigenin assay indicated that DangGui could inhibit high glucose-induced ROS in RMCs. These results suggest that DangGui has an ability to improve renal functions in STZ-diabetic rats through increasing endogenous BMP-7 expression and decreasing oxidative stress in kidney. The present study suggest that DangGui could be applied to improve renal functions in diabetic disorders.

## 1. Introduction

Many diabetic patients developed nephropathy and/or end-stage renal disease (ESRD) and the prevalence has progressively increased in recent [1]. Hyperglycemia is introduced as the main factor to induce diabetic nephropathy (DN). Clinical strategies for DN include glycemic control and blood pressure regulation [2, 3] while the therapeutic effect is not satisfied.

DangGui is the mandarin of dried root of *Angelica sinensis*. It is widely used in traditional Chinese medicine (TCM) to promote blood circulation and enrich blood, which usually means to increase the number of blood cells with improving their function. DangGui increases the secretion of many hematopoietic factors, such as interleukins, EPO, TPO, GM-CSF, TNF-*α* and IFN-*γ* from cells [4, 5]. DangGui could induce the proliferation of murine bone marrow mononuclear cells by activating ERK1/2 and P38 MAPK pathway [6]. DangGui also showed antiproliferative and pro-apoptotic activities in hepatic stellate cells (HSC-T6) [7]. In TCM, DangGui (*Angelica sinensis*) and Huang Qi (*Astragalus membranaceus*) are traditionally used along with each other [8]. The mixture of DangGui and Huang Qi is named as DangGui-Bu-Xue-Tang (GQM) that could alleviate the progression of DN in streptozotocin (STZ)-induced diabetic rats probably due to the lowering of angiotesin II and TGF-*β* mRNA [9, 10]. The antifibrotic action of GQM has been compared with an ACE inhibitor, enalapril, in puromycin-induced acute nephrosis animal model. The results showed that GQM could decrease proteinuria and BUN in addition to the lower expressions of TGF-*β*, collagen types III and IV, laminin and fibronectin [11]. In this study, we examined whether DangGui could show the renal protective effect. 

Bone morphogenetic protein-7 (BMP-7) could reduce glomerular and tubulointerstitial fibrosis and prevent the pathogenesis in diabetic nephropathy [12–16]. BMP-7 improves renal damage from hyperglycemia-induced oxidative stress in the kidney of diabetics [13, 14]. We also observed that control of hyperglycemia may improve renal function and reverse renal BMP-7 expressions at the initial stage of DN but not at the late stage of DN in type-1-like diabetic rats [12]. Hyperglycemia causes oxidative stress in renal cell that is implicated in the development of DN [17]. Endogenous BMP-7 could be raised by an anti-oxidant to activate the receptors for renal protection against the damage from high glucose (HG) [14]. The role of endogenous BMP-7 in the protection of DN seems important. However, the role of BMP-7 in the renal improvement effect of DangGui remains obscure. The present study is designed to investigate the effect of DangGui on renal functions and BMP-7 expression in STZ-diabetic rats. *We also determined the effect of DangGui on reactive oxygen species (ROS) generated from hyperglycemia*.

## 2. Methods

### 2.1. DangGui Preparation

The concentrated granules of DangGui, made of 98% pure authentic Chinese herbs of highest qualities, are produced by Han-Sheng Pharmtech, Inc. (Ping-Tong City, Taiwan) under internationally certified Good Manufacturing Practices guidelines. The macroscopic and microscopic examinations as well as thin-layer chromatography and high-performance liquid chromatography were used to identify the plants. The reference specimens were deposited at the herbarium of the supplier.

### 2.2. Animal Model for Diabetic Nephropathy

Six-week-old male Wistar rats, 180–200 g in weight, were obtained from the Animal Center of National Cheng Kung University Medical College. STZ-diabetic rats were induced by intravenously (i.v.) injecting STZ (65 mg/kg) (Sigma-Aldrich Inc., St Louis, MO, USA) into fasting rats as described previously [18]. Animals were considered to be diabetic if they had elevated plasma glucose concentrations of 350 mg/dL. All experiments were carried out at 9 weeks after the STZ-DN induction. STZ-diabetic rats were considered to have diabetic nephropathy (DN) when they had elevated levels of BUN and creatinine. All animal procedures were performed according to the guide for the care and use of laboratory animals of the National Institutes of Health. STZ-diabetic rats were treated with 5 mg/kg of DangGui or 1 mg/kg of ramipril twice a day for 1 or 4 weeks. The STZ-diabetic rats were divided into four groups (*n* = 6), respectively: vehicle-treated normal rats; vehicle-treated STZ-diabetic rats; DangGui-treated STZ diabetic rats; ramipril-treated STZ-diabetic rats.

### 2.3. Blood Glucose and Renal Functions Determination

Blood samples were collected from the rat femoral vein before the treatment and 1 h after the last treatment for estimating the levels of plasma glucose, urea nitrogen (BUN) and creatinine. The body weights were monitored during the experiment. At the end of the treatment, animals were killed and the kidneys were dissected, washed with saline, weighted for analysis and frozen in liquid nitrogen and then stored at −80°C for further analysis. Blood samples from rats were centrifuged at 12 000 g for 3 min. We incubated samples with glucose, BUN or creatinine kit reagents (AppliedBio assay kits; Hercules, CA, USA). The levels of blood glucose, BUN and serum creatinine were then estimated by the auto-analyzer (Quik-Lab, Ames, Miles Inc., Elkhart, Indiana, USA) run in duplicate.

### 2.4. DPPH Radical Scavenging Assay

The antioxidant activity of DangGui and ascorbic acid (vitamin C) was measured in terms of 1,1-diphenyl-2-picrylhydrazyl (DPPH) (Sigma-Aldrich Inc., St Louis, MO, USA) free radical scavenging ability, with slight modification. Vitamin C was used as a reference compound. The highest tested concentration of Vitamin C was considered as 100% of scavenging activity. A solution of DangGui at different concentrations was placed in a cuvette and 1 mL of 23.7 *μ*g/mL methanol solution of DPPH radical was added followed by incubation for 30 min. The decrease in absorbance at 517 nm was determined with a spectrophotometer. All determinations were performed in three replicates. The percentage inhibition of DPPH radical by the samples was calculated according to the following formula:

(1) $$\text{Percentage}\;\text{of}\;\text{scavenging}\;\text{of}\;\text{DPPH}=\left[1-\frac{A_{\left(s\right)}}{A_{\left(c\right)}}\right]\times 100,$$

where *A*_(*c*)_ is the absorbance of the control (without sample) and *A*_(*s*)_ is the absorbance of the sample at *t* = 30 min.

### 2.5. RMCs Cultures

The cell line, rat mesangial cells (RMCs), was purchased from the American Type Culture Collection (Manassas, VA, USA). RMCs were cultured in Dulbecco's modified Eagle's medium (DMEM; Gibco-BRL, Gaithersburg, MD, USA) containing 5 mmol/L glucose supplemented with 15% FBS and antibiotics at 37°C in 95% air, 5% CO_2_. We added 25 mmol/L glucose (final concentration 30 mmol/L) into serum-free DMEM for a HG medium. We use DMEM without FBS and additional glucose as a control medium. After ~60% confluent monolayer, the culture medium was displaced by control or HG medium with different treatments and then cultured for 24 h. DangGui (200 *μ*g/mL) and tiron (10 mmol/L; 4,5-dihydroxyl-1,3-benzene disulfonic acid; Sigma-Aldrich Inc., St Louis, MO, USA) were used.

### 2.6. Small Interfering RNA of BMP-7 Transfection

Duplexed RNA oligonucleotides for rat BMP-7 (Stealth RNAi TM) were synthesized by Invitrogen (Carlsbad, CA, USA). RMCs were transfected in combination with 40 pmol/L of BMP-7-specific small interfering RNA (siRNA) or scramble siRNA using Lipofectamine 2000 (Invitrogen) according to the manufacturer's protocols. The sequences of the BMP-7 Stealth RNAi (Bmp7-RSS340413) are (sense strands, 5′–3′) UGA UGUCAAAUACCAACCAGCCCU C and (antisense strands, 5′–3′) GAGGGCUGGUUGGUAUUUGACAUCA. After 12-h incubation in the transfection conditional medium for maximum target gene inhibition, it was changed to the regular culture medium with DangGui (200 mg/mL) for 24 h and analyzed.

### 2.7. Intracellular ROS Detection

The 10 000 of RMCs were seeded on 12-well cell culture plates. After 24 h growth, RMCs were placed in control or HG medium and treated with tiron (10 *μ*mol/L) or DangGui (200 *μ*g) for further 24-h incubation and then harvested. The RMCs were fixed with 3.7% paraformaldehyde. Thirty minutes after cell fixation and three times wash with PBS, 5 *μ*mol/L of dihydroethidium (DHE; Invitrogen) was added as a fluorescent indicator of ROS generated in response to the described treatment. Images were collected with an Olympus IX70 fluorescence microscope fitted with an Olympus America camera and MagnaFire 2.1 software.

### 2.8. Measurement of Superoxide Generation

Superoxide production was determined by the lucigenin method. RMCs were incubated in the NG (control) or HG medium for 24 h in the absence or presence of various treatments described above. Then, RMCs were trypsinized and collected by centrifugation, and the pellet was washed in the modified Krebs buffer containing NaCl (130 mmol), KCl (5 mmol), MgCl_2_ (1 mmol), CaCl_2_ (1.5 mmol), K_2_HPO_4_ (1 mmol) and HEPES (20 mmol), pH 7.4. After washing, the RMCSs were resuspended in the Krebs buffer with 1 mg/mL BSA, and the cell concentration was adjusted to 1 × 10^7^ in 900 *μ*L buffer. To measure ROS production, the cell suspension was transferred into the measuring chamber and assessed in a Chemiluminescence Analyzer (Tohoku Electronic Industrial Co., Ltd, Japan). Measurement was started by an injection of 100 *μ*l lucigenin (final concentration, 4 × 10^−4^ mmol/L). Photon emission was counted every 10 s for up to 10 min.

### 2.9. Western Blot Analysis for Kidney and RMCs

BMP-7 expressions in rat kidney or RMCs were determined by western blotting analysis. We use the RIPA buffer to extract total protein. For western analysis, proteins were separated by SDS-PAGE, transferred and immobilized on a nitrocellulose membrane. The membrane was blocked with 5% non-fat dry milk in phosphate buffered saline containing 0.1% Tween 20 (PBS-T) for 2-h incubation at room temperature. The membrane was then washed in PBS-T and hybridized with primary antibodies diluted to a proper concentration in PBS-T for 16 h. Specific antibodies (Santa Cruz Biotechnology Inc., Santa Cruz, CA, USA) for BMP-7 (diluted in 1 : 200) and *β*-actin (diluted in 1 : 1000) were used. Incubation with secondary antibodies and detection of the antigen-antibody complex were performed using the ECL kit (Amersham Biosciences, UK). Densities of the obtained immunoblots were quantified using a laser densitometer.

### 2.10. Statistical Analysis

Results were expressed as mean ± SE. Statistical analysis was carried out by using ANOVA analysis and Newman-Keuls *post* *hoc* analysis. Statistical significance was achieved if *P* < 0.05 or *P* < 0.001.

## 3. Results

### 3.1. Effect of DangGui on Renal Functions in STZ-Diabetic Rats

**Figure 1:**
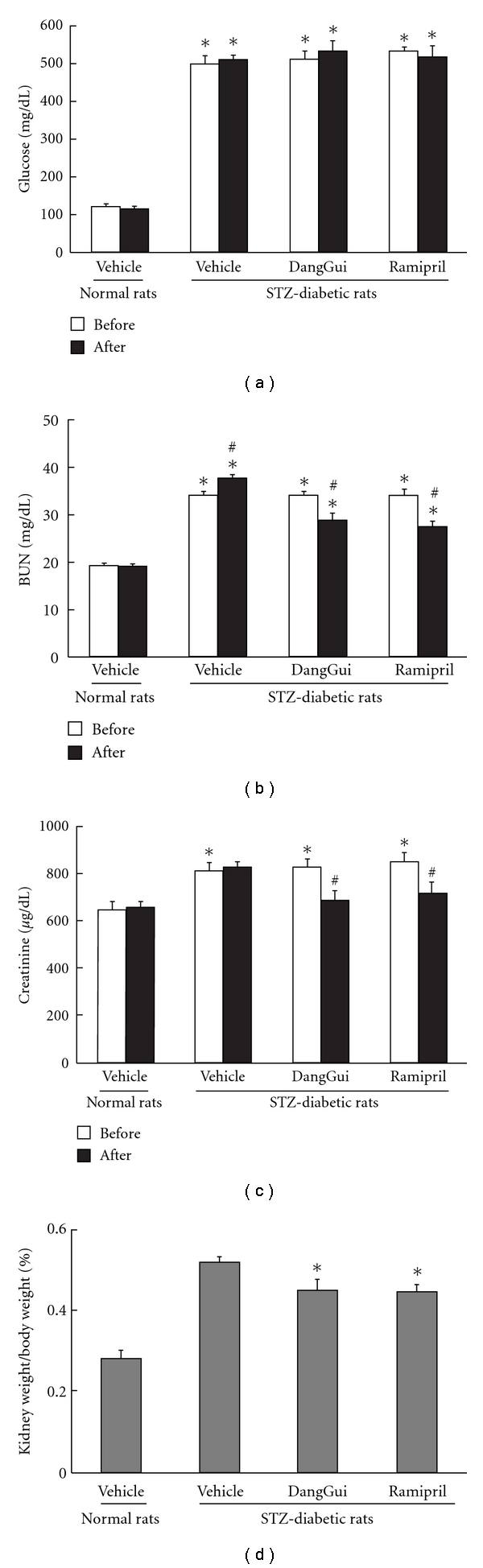
Effects of DangGui on the levels of blood glucose (a), BUN (b), creatinine (c) and kidney/body weight ratio (d) in STZ-diabetic rat after 1-week DangGui (5 mg/kg) or ramipril (1 mg/kg) treatment. *P < 0.05 values of STZ-diabetic rats compared to the value of vehicle-treated normal rats. ^#^ *P* < 0.05 the significance for values of after treatment compared to treatment before.

**Figure 2:**
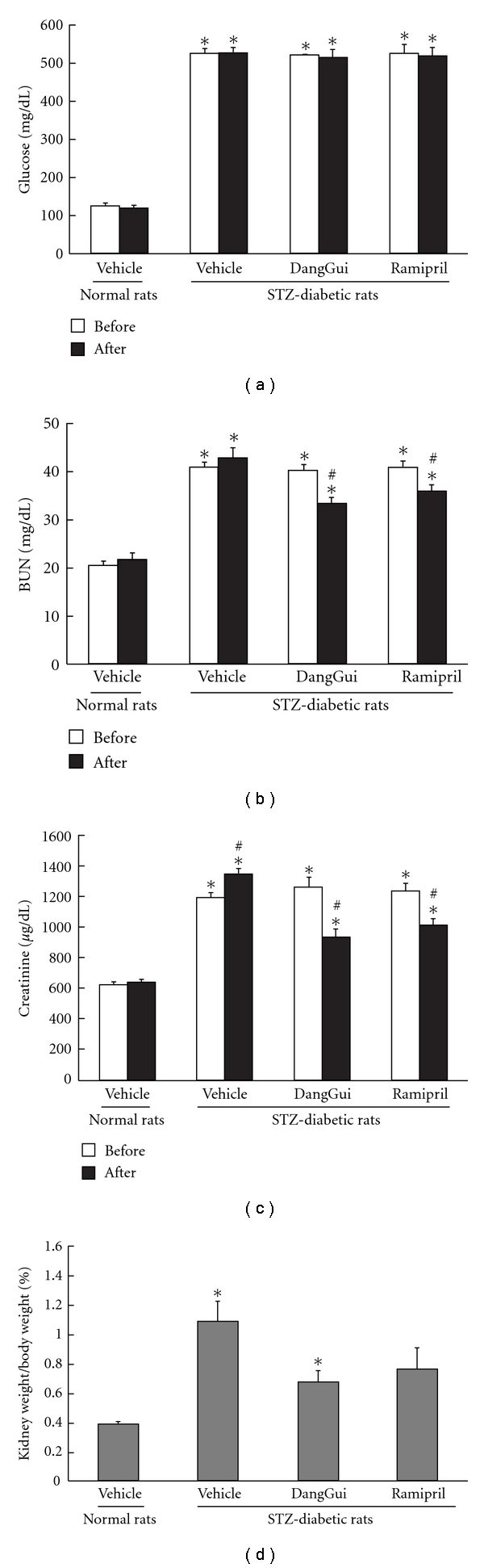
Effects of DangGui on the levels of blood glucose (a), BUN (b), creatinine (c) and kidney/body weight ratio (d) in STZ-diabetic rat after 4-week DangGui (5 mg/kg) or ramipril (1 mg/kg) treatment. The different treatments on rats are described in the Materials section. *P < 0.05 values of STZ-diabetic rats compared to the value of vehicle-treated normal rats. ^#^ *P* < 0.05 is the significance for values after treatment compared to treatment before.

Wistar rats were injected with STZ to induce type-1 like diabetic animal. After 9 weeks, the serum levels of glucose, BUN, creatinine and kidney weight in STZ-diabetic rats were significantly higher than normal rats (*P* < 0.05) (Figure 1). We treated angiotensin-converting enzyme inhibitor, ramipril [19], to STZ-diabetic rats as positive control. After treatment with DangGui or ramipril for 7 days, the serum levels of BUN and creatinine and kidney weight in STZ-diabetic rats were effectively reversed but these effects were not shown on glucose (Figures 1(a)–1(d)). The serum levels of BUN and creatinine in 4-week vehicle-treated STZ-diabetic rats (Figures 2(b) and 2(c)) were higher than those in 1-week vehicle-treated STZ-diabetic rats (Figures 1(b) and 1(c)). Four-week treatment of DangGui or ramipril could improve the renal functions and decrease kidney weight of STZ-diabetic rats (Figures 2(a)–2(d)).

### 3.2. Recovery of BMP-7 in Kidney and RMCs by DangGui

**Figure 3:**
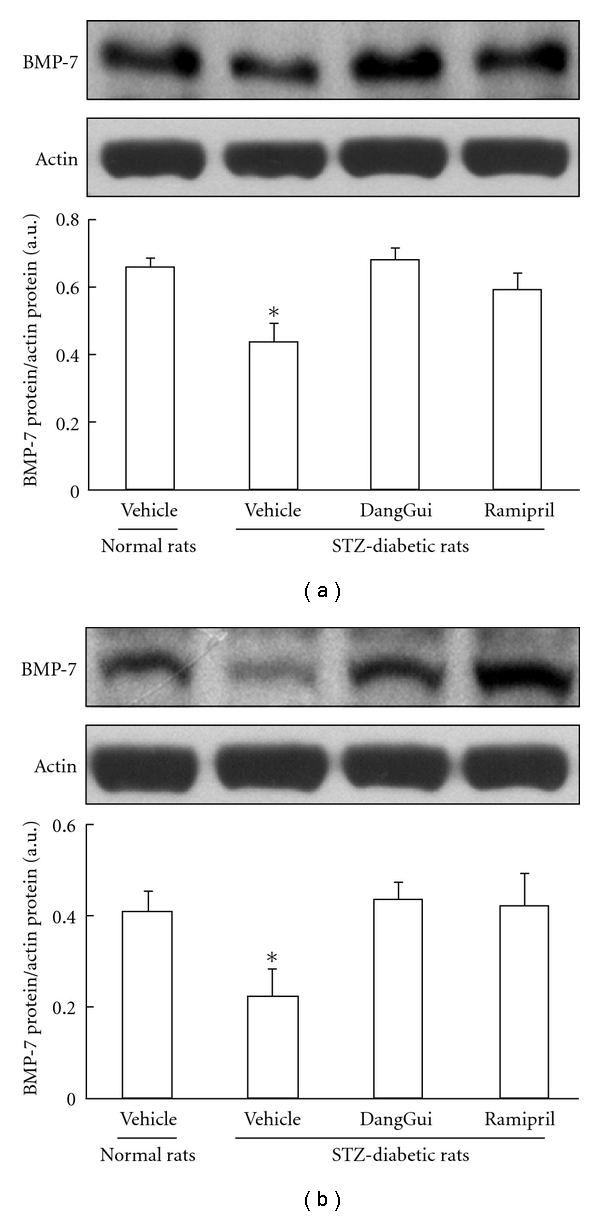
Change of the BMP-7 level in the kidney of STZ-diabetic rat after 1-week (a) or 4-week (b) treatment of DangGui (5 mg/kg) or ramipril (1 mg/kg). Western blotting analysis of BMP-7 expression was carried out in the kidney of 9-week STZ-diabetic rat. Upper picture shows the protein level of BMP-7 or *β*-actin in kidney isolated from STZ-diabetic rat. The different treatments on rats are described in the Materials section. Quantification of protein level using BMP-7/*β*-actin expressed as mean ± SE (*n* = 4 per group) in each column is indicated in the lower panel. *P < 0.05 compared to the value of vehicle-treated normal rats.

Changes of renal BMP-7 expression of STZ-diabetic rats were analyzed using western blot. BMP-7 expression in the kidney of STZ-diabetic rats was reduced in a way related to diabetic induction (35% decreased in 9-week STZ-diabetic rats and 50% decreased in 13-week STZ-diabetic rats as compared to normal rats) (Figures 3(a) and 3(b)). After DangGui or ramipril administration, BMP-7 expressions in the kidney were increased in both 1- and 4-week-treated STZ-diabetic rat (Figures 3(a) and 3(b)).

**Figure 4:**
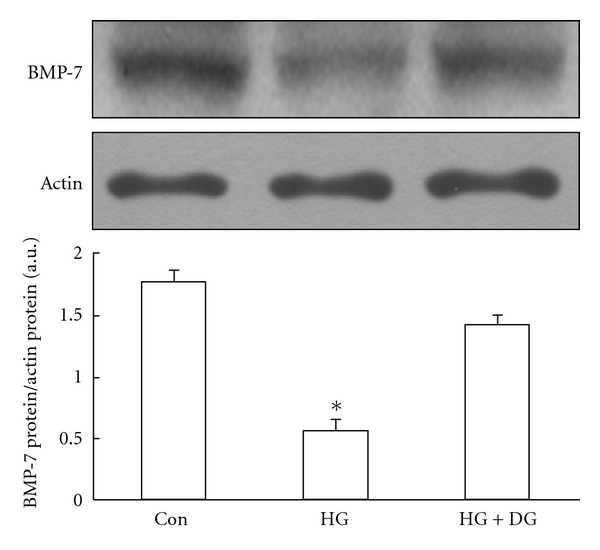
Effect of DangGui on the BMP-7 level in high-glucose-treated RMCs. RMCs were cultured in normal glucose (5 mmol/L; Con) or high-glucose (30 mmol/L; HG) medium in the presence of 200 *μ*g/mL DangGui (DG) for 24 h incubation. Western blot analysis of BMP-7 expression in RMCs. Upper picture shows the protein level of BMP-7 or *β*-actin in RMCs. The conditional treatments on RMCs are described in the Materials and methods section. Quantification of protein level using BMP-7/*β*-actin expressed as mean ± SE (*n* = 4 per group) in each column is indicated in the lower panel. *P < 0.05 compared to the value of control medium.

**Figure 5:**
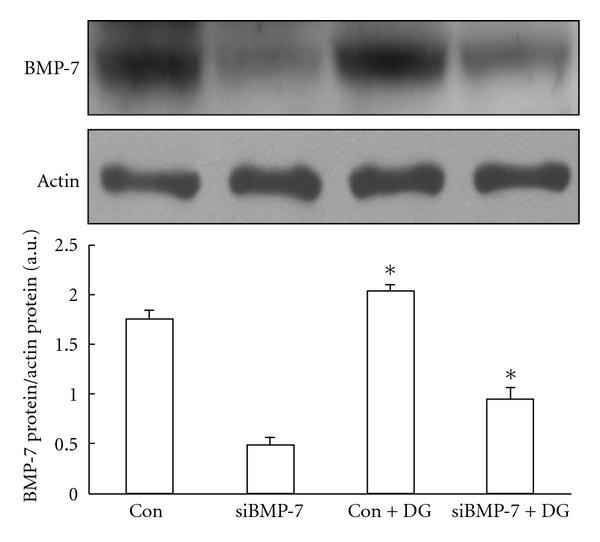
Effect of DangGui on BMP-7 protein in RMCs-treated BMP-7 siRNA. RMCs were cultured in scramble siRNA (Con) or BMP-7 siRNA (siBMP-7) transfection conditional medium for 12 h. RMCs were cultured in normal glucose (5 mmol/L) medium in the presence of 200 *μ*g/mL DangGui (DG) after being transfected with scramble siRNA (Con+DG) or BMP-7 siRNA (siBMP-7+DG) for further 24 h incubation. Western blot analysis of BMP-7 expression in RMCs. Upper picture shows the protein level of BMP-7 or *β*-actin in RMCs. The conditional treatments on RMCs are described in the Materials section. Quantification of protein level using BMP-7/*β*-actin expressed as mean ± SE (*n* = 4 per group) in each column is indicated in the lower panel. *P < 0.05 compared to the value after DangGui treatment.

Then, we confirmed the direct effect of DangGui on BMP-7 expression *in vitro*. In the preliminary tests (data not shown), we found that BMP-7 expression in RMCs was reduced markedly by high glucose incubation after 24 h but it could not be increased by DangGui within 12 h. Thus, we investigated the effect of DangGui on BMP-7 expression in RMCs after the 24-h incubation. The BMP-7 expression in high glucose-treated RMCs was markedly decreased and it can be reversed by DangGui treatment for 24 h (Figure 4). The small interference RNA of BMP-7 (siBMP-7) and scramble RNAi were then employed to identify the role of BMP-7 in this action of DangGui. The level of BMP-7 protein expression was markedly reduced after siBMP-7 transfection in RMCs cultured in the normal medium. But the same siBMP-7 transfection led to cell death in RMCs incubated with the high glucose medium. Thus, we have to give up the study in the high glucose medium. The BMP-7 expression of RMCs was inhibited by over 75% after 12-h siBMP-7 transfection comparing to scramble RNAi transfection (Figure 5, lanes 1 and 2). After 12-h transfection, we treated DangGui to those RMCs and incubated for further 24 h. The results showed that DangGui could increase the BMP-7 expression in scramble RNAi-transfected RMCs (Figure 5 and lane 3). The decreased BMP-7 protein by siBMP-7 transfection could be reversed by DangGui treatment for 24 h (Figure 5 and lane 4).

### 3.3. DangGui Decreased Reactive Oxygen Species (ROS)

**Figure 6:**
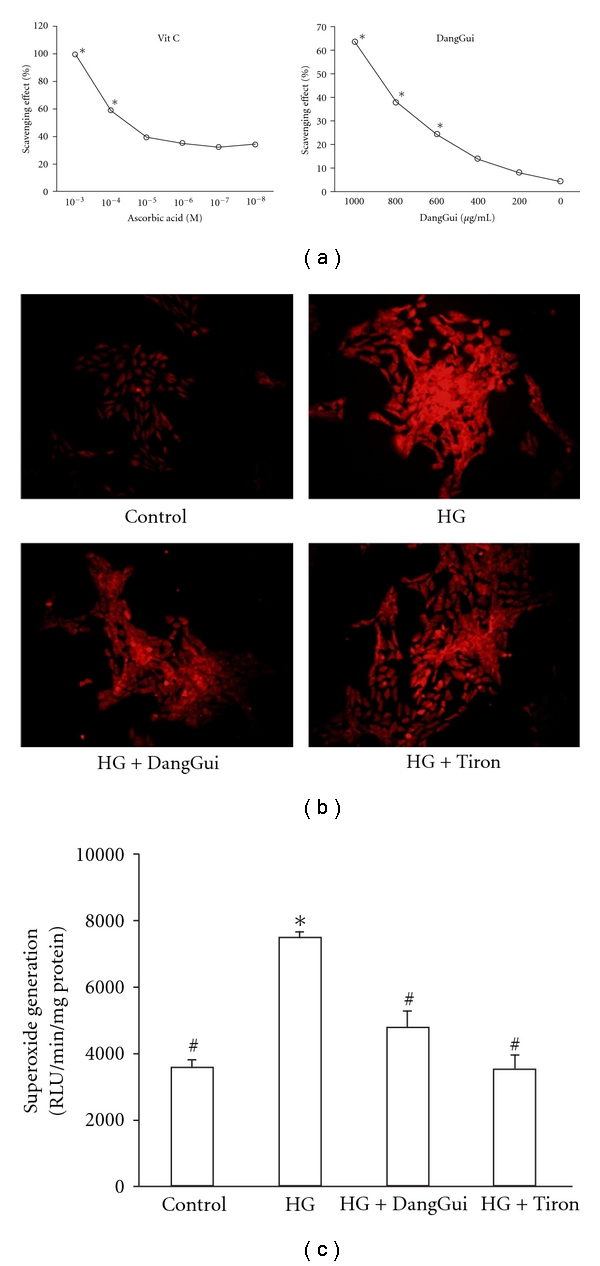
Effect of DangGui on free radicals in glucose-exposed RMCs. (a) Direct free radical scavenging activity of DangGui was determined by the DPPH radical scavenging assay. Values represent the data of three independent experiments (SE < 0.05). Ascorbic acid was used as a reference compound. (b) DHE stains were used to visualize the intracellular ROS in RMCs. (c) Lucigenin assays were used to quantitate the generation of superoxide in RMCs. RMCs were cultured in normal glucose (5 mmol/L; control) or high glucose (30 mmol/L; HG) medium in the presence of DangGui (200 *μ*g/mL) or tiron (10 *μ*mol/L) for 24-h incubation. *P < 0.05 compared to control, ^#^ *P* < 0.05 compared to HG.

The direct ROS scavenging effect of DangGui was evaluated using the DPPH radical scavenging assay.  Figure 6(a) showed that DangGui could scavenge the free radicals directly in a concentration-dependent manner similar to vitamin C.

### 3.4. DangGui Decreases High-Glucose-Induced ROS in RMCs

In the previous study [14], ROS was characterized to be raised in RMCs incubating with a HG medium and this can be reduced by rhBMP-7. The antioxidative ability of DangGui was visualized by DHE stain (Figure 6(b)) and measured by the lucigenin assay (Figure 6(c)). We observed that ROS increased in RMCs by HG was markedly inhibited by DangGui in a way similar to tiron. The lucigenin assay revealed that BMP-7 could lower the high glucose-induced superoxide generation in RMCs.

## 4. Discussion

In present study, we found that DangGui improved the renal functions and increased BMP-7 expression in the kidney of STZ-diabetic rat. DangGui could directly increase endogenous BMP-7 and decrease ROS induced by HG in RMCs. We used STZ to induce type-1 like diabetic rats as described previously [18]. Development of DN is widely characterized using the higher plasma levels of creatinine and BUN in STZ-induced diabetic rats. In our study, the renal functions of the STZ-induced diabetic rats showed typical features of DN as described previously [20, 21]. Following the previous reports [22], 1-week-treated STZ-induced diabetic rats belong to the initial stage of DN and 4-week-treated STZ-induced diabetic rat are defined as the established stage and/or late phase of DN. Both stages of DN were improved by DangGui treatment in the present study.

BMP-7 is observed to express mostly in adult mammalian kidney to help maintain renal structure and physiological function while BMP-7 expression decreased in damaged kidney [22]. Exogenous BMP-7 is mentioned to improve renal function and prevent glomerular sclerosis in diabetic rats [23, 24]. However, BMP-7 fails to attenuate the protein overload-induced renal interstitial fibrosis [24]. Thus, the reserve of endogenous BMP-7 in the kidney to prevent injuries seems to be pivotal. We found that renal BMP-7 expression was lowered in both 1- or 4-week vehicle-treated STZ-diabetic rats. After 1-week treatment, DangGui improved renal functions and increased BMP-7 expression in type-1 like diabetic rats. These actions of DangGui were more effective in 4-week-treated diabetic rats than in 1-week-treated diabetic rats. To understand the changes of BMP-7 in the kidney of rats with DN, we employed the cultured mesangial cells (RMCs) to mimic the *in vivo* changes. Hyperglycemia causes oxidative stress and leads to increased levels of transforming growth factor-*β* (TGF-*β*) and increased production of extracellular matrix (ECM) proteins. This increased production in glomerular mesangial cells has been implicated in the development of diabetic nephropathy (DN) [16]. *In vitro* studies have shown that mesangial cells are the major source of free radicals after exposure to high glucose concentrations [3, 25]. Thus, the cultured rat mesangial cells incubated in the high glucose medium can be used as an *in vitro* model in order to investigate diabetic nephropathy in this study. In cultured cell, endogenous BMP-7 in RMCs was reduced after 24-h high glucose exposure. When endogenous BMP-7 was reduced by siRNA, as described previously, RMCs lost their resistance to HG-generated ROS. However, this effect can be reversed with rhBMP-7 treatment [14]. It was suggested that BMP-7 has an antioxidative activity. As shown in Figure 4, the decrease of BMP-7 by high glucose incubation was reversed by DangGui. However, RMCs transfected with BMP-7 RNAi were all expired in the high glucose medium although scramble RNAi-transfected RMCs remained alive under HG in the preliminary experiment. Thus, BMP-7 seems essential in the growth of RMCs under HG. Otherwise, we knock down BMP-7 expression in RMCs using BMP-7 RNAi successfully in the normal medium (Figure 5). After 12-h transfection with scramble or BMP-7 RNAi, we replaced the transfection conditional medium with the regular culture medium containing DangGui to culture for further 24 h. We observed that DangGui increased BMP-7 protein in RMCs transfected with either scramble RNAi or siBMP-7. One of the possible reasons for the increase of BMP-7 expression by DangGui in siBMP-7-transfected RMCs may be related to the longer lasting time of DangGui (24 h) as compared to siBMP-7 (12 h). Nevertheless, the direct activation of BMP-7 expression by DangGui could be considered. 

**Figure 7:**
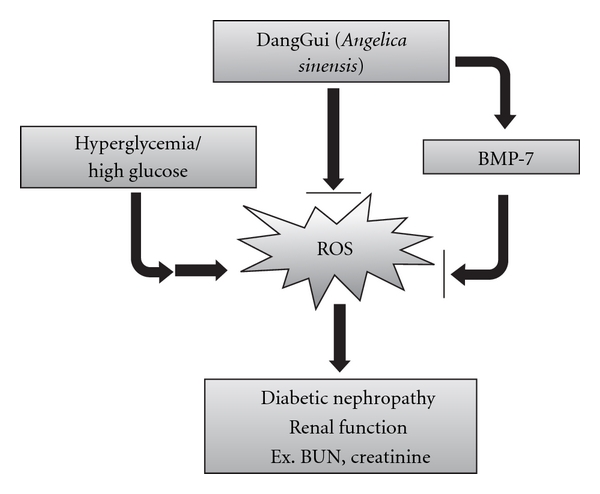
Hypothesis for the merit of DangGui in decrease of ROS through a direct effect and/or an increase of BMP-7 expression in diabetic nephropathy.

Moreover, DangGui improved the renal functions and increased BMP-7 expression as the dose failed to modify blood glucose level in STZ-diabetic rats. It has been documented that glucose stimulates excessive production of ROS leading to DN [25]. High glucose-induced ROS is one of the main reasons for the decrease of BMP-7 expression in the kidney of STZ-diabetic rats and/or high glucose-exposed RMCs [25]. DangGui was reported as a native free radical scavenger [26]. There is no report showing the anti-oxidative stress of DangGui in diabetic nephropathy. However, some studies indicated that DangGui has neuroprotective activity through scavenging free radicals, such as Z-ligustilide from DangGui protected against H_2_O_2_-induced cytotoxicity in PC12 cells and forebrain I/R by enhancing antioxidant defense [27, 28]. Also, coniferyl ferulate is the main antioxidant from the essential oil of DangGui [29] and ferulic acid could reduce neuronal damage from exposure-free radicals [30, 31]. Moreover, the polysaccharides of DangGui may protect macrophages against tert-butylhydroperoxide-induced oxidative injury [32, 33]. These reports indicated that DangGui could protect tissues from oxidative stress. We found that the action of DangGui in STZ-diabetic rats seems to be related to the decrease of ROS. The DPPH assay revealed that DangGui has an ability to scavenge the free radical directly. DHE stain and lucigenin assay also showed that DangGui can decrease high glucose-induced ROS in a way as tiron, the famous ROS scavenger. Taken together, we suggested that DangGui could decrease ROS in the kidney of diabetic rats or in mesangial cells generated by HG. In Figure 7, we summarized this view that DangGui could increase renal BMP-7 to prevent the kidney from damage of oxidative stress produced in diabetic disorders.

In conclusion, DangGui could prevent renal functions through an increase of endogenous BMP-7 expression and a direct reduction of ROS. DangGui could be applied for handling of diabetic nephropathy base on its safety after long used history in TCM.
